# Single cell metabolic imaging of tumor and immune cells *in vivo* in melanoma bearing mice

**DOI:** 10.3389/fonc.2023.1110503

**Published:** 2023-03-20

**Authors:** Alexa R. Heaton, Peter R. Rehani, Anna Hoefges, Angelica F. Lopez, Amy K. Erbe, Paul M. Sondel, Melissa C. Skala

**Affiliations:** ^1^ Morgridge Institute for Research, Madison, WI, United States; ^2^ Department of Human Oncology, University of Wisconsin, Madison, WI, United States; ^3^ Department of Biomedical Engineering, University of Wisconsin, Madison, WI, United States; ^4^ Department of Pediatrics, University of Wisconsin, Madison, WI, United States

**Keywords:** intravital imaging, immune cells, melanoma, metabolism, murine models, multiphoton/two-photon imaging, autofluorescence

## Abstract

**Introduction:**

Metabolic reprogramming of cancer and immune cells occurs during tumorigenesis and has a significant impact on cancer progression. Unfortunately, current techniques to measure tumor and immune cell metabolism require sample destruction and/or cell isolations that remove the spatial context. Two-photon fluorescence lifetime imaging microscopy (FLIM) of the autofluorescent metabolic coenzymes nicotinamide adenine dinucleotide (phosphate) (NAD(P)H) and flavin adenine dinucleotide (FAD) provides *in vivo* images of cell metabolism at a single cell level.

**Methods:**

Here, we report an immunocompetent mCherry reporter mouse model for immune cells that express CD4 either during differentiation or CD4 and/or CD8 in their mature state and perform *in vivo* imaging of immune and cancer cells within a syngeneic B78 melanoma model. We also report an algorithm for single cell segmentation of mCherry-expressing immune cells within *in vivo* images.

**Results:**

We found that immune cells within B78 tumors exhibited decreased FAD mean lifetime and an increased proportion of bound FAD compared to immune cells within spleens. Tumor infiltrating immune cell size also increased compared to immune cells from spleens. These changes are consistent with a shift towards increased activation and proliferation in tumor infiltrating immune cells compared to immune cells from spleens. Tumor infiltrating immune cells exhibited increased FAD mean lifetime and increased protein-bound FAD lifetime compared to B78 tumor cells within the same tumor. Single cell metabolic heterogeneity was observed in both immune and tumor cells *in vivo*.

**Discussion:**

This approach can be used to monitor single cell metabolic heterogeneity in tumor cells and immune cells to study promising treatments for cancer in the native *in vivo* context.

## 1 Introduction

Metabolic reprogramming is a hallmark of cancer (1, 2). Cancer cells increase their metabolic activity to fuel rapid cell proliferation. At the same time, fast tumor growth leads to poor vascularization, decreased oxygen availability, increased metabolic waste products, and increased acidity in the tumor microenvironment (TME). To survive, cancer cells increase their metabolic plasticity and adapt to the available nutrients. Whereas healthy cells primarily metabolize glucose using oxidative phosphorylation, cancer cells primarily metabolize glucose using glycolysis (3–7). Immune cells also undergo metabolic changes during tumorigenesis that correlate to immune cell phenotype and function (1, 8, 9). Some immune cell reprogramming is critical for increased proliferation, activation, and cytokine release while other reprogramming is necessary to compete with cancer cells for nutrients in the TME. For example, many lymphocytes shift toward increased glycolysis upon stimulation to support proliferation and cytokine release, however, nutrient competition in the TME is fierce as cancer cells also increase glucose uptake (1, 2). This nutrient competition can lead to detrimental effects on the immune cells such as exhaustion or dysfunction, which further promote tumorigenesis. Improved understanding of tumor and immune cell metabolism and their complex interplay in the TME will improve clinical outcomes in cancer. This knowledge could guide the development of therapies aimed at modifying metabolism to limit tumor cell metabolism and promote healthy immune cell metabolism.

Current techniques to measure tumor and immune cell metabolism require destruction of the sample and/or single cell isolations that remove relevant spatial context and may limit collection of rare cell populations. There is a need for methods that can monitor metabolic changes within intact *in vivo* samples that maintain the native TME. Intravital optical metabolic imaging (OMI) resolves many of these shortcomings by providing *in vivo*, label free, single cell imaging of metabolic changes. These metabolic changes are quantified with the fluorescence intensities and lifetimes of metabolic coenzymes NAD(P)H and FAD, which are autofluorescent molecules present in all cells (10–12). The fluorescence of NADH and NADPH are difficult to separate spectrally, so their combined fluorescence will be referred to here as NAD(P)H. These metabolic coenzymes shuttle electrons during most metabolic processes, where NAD(P)H is an electron donor and FAD is an electron acceptor. NAD(P)H and FAD intensity changes are often quantified using the optical redox ratio, defined here as the intensity of NAD(P)H divided by the sum of the intensity of NAD(P)H plus FAD, which monitors changes in the oxidation-reduction state of the cell (10–13). A decrease in optical redox ratio here indicates a more oxidized environment. The fluorescence lifetime, the time a molecule remains in its excited state before relaxing back to ground state, informs on the protein binding activity of NAD(P)H and FAD as well as changes in the cellular microenvironment. The fluorescence lifetimes of free versus protein-bound NAD(P)H and FAD are distinct and can be quantified using fluorescence lifetime imaging microscopy (FLIM) (11–15). FLIM of NAD(P)H and FAD reports on changes in free and protein-bound lifetimes due to microenvironmental factors and preferred binding partners, while also providing relative proportions of free and protein-bound pools. Previous studies have illustrated that OMI can quantify tumor cell (16–23) or immune cell (24–29) metabolic changes separately. However, imaging of *in vivo* metabolic changes in immune cells between different tissues of origin and uncoupling tumor cell and immune cell metabolism within the same tissue has not been well studied in the context of melanoma.

Here we demonstrate *in vivo* optical metabolic imaging, a fluorescent reporter mouse model, and single cell segmentation approaches to quantify immune cell and tumor cell metabolism *in vivo*. As proof-of-principle for this method, we show that: 1) immune cell metabolism within spleens differs from metabolism of tumor infiltrating immune cells, 2) tumor cells and tumor infiltrating immune cells also differ metabolically, and 3) single cell metabolic heterogeneity is present in both immune and tumor cells populations *in vivo*. Overall, this approach can be used to monitor single cell metabolic heterogeneity in tumor cells and immune cells to study promising treatments for cancer in the native *in vivo* context.

## 2 Materials and methods

### 2.1 CD4 mCherry reporter mouse breeding, tumor inoculation, and surgery

Animals were housed and treated under an animal protocol approved by the Institutional Animal Care and Use Committee at the University of Wisconsin-Madison. The CD4 mCherry reporter mouse model was made using Cre-*lox* methods by crossing CD4-Cre mice (Jax 022071) with floxed H2B-mCherry mice (Jax 023139) at the University of Wisconsin Biomedical Research Model Services (**Figure S1**) (30, 31). H2B-mCherry homozygous/CD4cre hemizygous mice were successfully bred and genotyped to confirm mCherry labeling to all CD4+ cells that expressed CD4 either during differentiation or in their mature state. The reporter mCherry was chosen to avoid spectral overlap with autofluorescence of NAD(P)H and FAD, and to confirm the identity of *in vivo* cells with CD4 expression presently or in their lineage (32–34).

B78-D14 (B78) melanoma is a poorly immunogenic cell line derived from B78-H1 melanoma cells, which were originally derived from B16 melanoma (35–37). These cells were obtained from Ralph Reisfeld (Scripps Research Institute) in 2002. B78 cells were transfected with functional GD2/GD3 synthase to express the disialoganglioside GD2 (36, 37), which is overexpressed on the surface of many human tumors including melanoma (38). These B78 cells were also found to lack melanin. B78 cells were grown in RPMI-1640 (Gibco) supplemented with 10% FBS and 1% penicillin/streptomycin, with periodic supplementation with 400 μg G418 and 500 μg Hygromycin B per mL. Mycoplasma testing was performed every 6 months. B78 tumors were engrafted by intradermal flank injection of 2×10^6^ tumor cells (39). We have previously developed successful immunotherapy regimens for mice bearing these B78 tumors, enabling the cure of mice with measurable tumors (~100 mm^3^ volume) (40–42). These cured mice have demonstrated tumor-specific T-cell mediated memory, as detected by rejection of rechallenge with the same vs. immunologically distinct tumors. Here, we continue our investigation of the B78 tumor model and expand our work into the metabolic field. Tumor size was determined using calipers and volume approximated as:


$$\text{tumor volume}=({\text{tumor width}}^{2}\times \text{tumor length})/2$$

**Figure 1:**
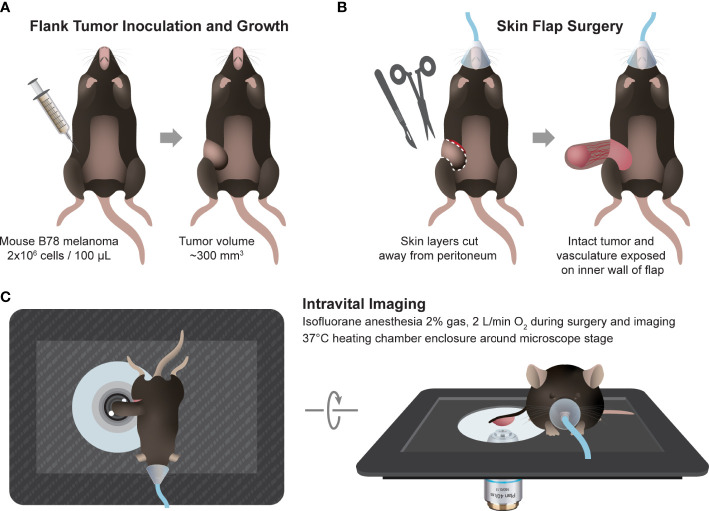
*In vivo* multiphoton imaging experimental workflow. **(A)** Workflow began with intradermal inoculation of 2×10^6^ B78 melanoma cells on mouse flank. Tumors were monitored weekly until they reached ~300 mm^3^ volume. **(B)** On the day of imaging, the mouse was anesthetized, and tumor skin flap surgery was performed where dermal and subcutaneous skin layers were gently cut away from the peritoneum revealing the tumor with intact vasculature. **(C)** The tumor and skin flap were placed on a glass slide for imaging and the mouse on a specially designed microscope stage. Throughout *in vivo* imaging, the mouse was kept under anesthesia and inside a heating chamber that enclosed the microscope stage.

Intravital imaging of the mouse tumors (n = 2) was performed in untreated mice when tumors were well established (280-340 mm^3^), 5-6 weeks after inoculation. Immediately prior to tumor imaging, skin flap surgery exposed flank tumors. Mice were anesthetized with isoflurane, then the skin around the tumor was cut into a flap and separated from the body cavity so that the tumor laid flat on the imaging stage while still connected to the vasculature (43–45). Mice were placed on a specialized microscope stage for imaging and kept in a heating chamber (air maintained at 37°C) during imaging. An imaging dish insert and PBS for coupling were used with surgical tape to secure skin flap tumors (**Figure 1**).

### 2.2 Multiphoton imaging

Intravital/*in vivo* imaging was performed live, as described in 2.1 above (**Figure 1**). *Ex vivo* imaging of whole spleens was performed immediately following intravital imaging and euthanasia. Excised spleens were secured to an imaging dish with PBS coupling and tape. *Ex vivo* imaging was completed within one hour post mouse euthanasia, accurately capturing splenic metabolism based on our previous work that showed that *ex vivo* metabolism is statistically identical to *in vivo* metabolism for up to 12 hours post euthanasia (46). *In vitro* imaging of CD3+ splenic T cells was performed following negative selection magnetic bead separation with an EasySep™ Mouse T Cell Isolation Kit (STEMCELL Technologies) and cells were plated on a glass imaging dish.

Autofluorescence images were captured with a custom-built multi-photon microscope (Bruker) using an ultrafast femtosecond laser (InSight DSC, Spectra Physics). Fluorescence lifetime measurements were performed using time-correlated single photon counting electronics (Becker & Hickl). Fluorescence emission, in FLIM mode, was detected simultaneously in three channels using bandpass filters of 466/40 nm (NAD(P)H), 514/30 nm (FAD), and 650/45 nm (mCherry) prior to detection with three GaAsP photomultiplier tubes (Hamamatsu). All three fluorophores were simultaneously excited using a previously reported wavelength mixing approach (47, 48) with two-photon excitation at 750 nm (tunable line, λ_1_) and 1041 nm (fixed line, λ_2_), and two-color two-photon excitation at 872 nm (λ_2c-2p_):


$$\lambda_{2c-2p}=\;\frac{2}{\frac{1}{\lambda_{1}}+\;\frac{1}{\lambda_{2}}}$$

Briefly, the laser source tuned to 750 nm (NAD(P)H excitation) was delayed and collimated with the secondary laser line fixed at 1041 nm (mCherry excitation) for spatial and temporal overlap at each raster-scanned focal point (2-color excitation of FAD with 750 nm + 1041 nm). During wavelength mixing in FLIM mode, typical power was 0.9-1.9 mW for the 750 nm laser and 0.7-1.0 mW for the 1041 laser. Second-harmonic generation (SHG) images, in galvo mode, of collagen fibers and mCherry-expressing immune cells were detected using bandpass filters of 514/30 nm (collagen/SHG) and 650/45 nm (mCherry) at 1041 nm excitation (typical power 2.7-3.0 mW). There will be some SHG contribution to the FAD channel from our 1041 nm laser, but we can distinguish SHG signal from true FAD signal due to the clear morphology and lifetime differences between collagen fibers and FAD-containing cells. Sum frequency generation (SFG) signal (436 nm) generated *via* our wavelength mixing setup is excluded using these bandpass filters (47, 48). A phasor plot was used to confirm that no mCherry signal was present in the FAD channel. All images were acquired with a 40×/1.13 NA water-immersion objective (Nikon) at 512×512 pixel resolution and an optical zoom of 1.0-2.0. A daily fluorescence standard measurement was collected by imaging a YG fluorescent bead (Polysciences Inc.) and verifying the measured lifetime with reported lifetime values. NAD(P)H and FAD intensity and lifetime images were acquired to sample metabolic behavior of B78 tumor and immune cells across 3-8 fields of view and multiple depths within each tissue.

### 2.3 Multiphoton image analysis

#### 2.3.1 Fluorescence lifetime fitting

The fluorescence lifetimes of free and protein-bound NAD(P)H and FAD are distinct, and these lifetimes along with their weights can be recovered with a two-exponential fit function. Therefore, fluorescence lifetime decays for both NAD(P)H and FAD were fit to the following bi-exponential function in SPCImage:


$$I\left(t\right)=\;\alpha_{1}e^{-t/\tau_{1}}+\;\alpha_{2}e^{-t/\tau_{2}}+C$$

For NAD(P)H, τ_1_ corresponds to the free lifetime, τ_2_ corresponds to the protein-bound lifetime, and the weights (α_1_, α_2_; α_1_ + α_2_ = 1) correspond to the proportion of free and protein-bound NAD(P)H, respectively (11, 14, 15). Conversely for FAD, τ_1_ corresponds to the protein-bound lifetime and τ_2_ corresponds to the free lifetime (13, 15, 49). An instrument response function was measured using SHG (900 nm excitation) from urea crystals for input into the decay fit procedure. The following fluorescence lifetime endpoints were calculated from the fitted model: τ_1_, τ_2_, α_1_, and α_2_ for both NAD(P)H and FAD; along with the optical redox ratio (**Table 1**) (10, 11).

**Table 1:** Optical metabolic imaging parameters.

Optical Metabolic Imaging Parameters	
NAD(P)H τ_1_	Lifetime free NAD(P)H – short
NAD(P)H α_1_	% free NAD(P)H
NAD(P)H τ_2_	Lifetime bound NAD(P)H – long
NAD(P)H α_2_	% protein bound NAD(P)H
NAD(P)H τ_m_	NAD(P)H mean lifetime = α_1_τ_1_+α_2_τ_2_
FAD τ_1_	Lifetime bound FAD – short
FAD α_1_	% protein bound FAD
FAD τ_2_	Lifetime free FAD – long
FAD α_2_	% free FAD
FAD τ_m_	FAD mean lifetime = α_1_τ_1_+α_2_τ_2_
Optical Redox Ratio	= $\frac{\text{Intensity NAD}\left(\text{P}\right)\text{H}}{\text{Intensity NAD}\left(\text{P}\right)\text{H}+\text{Intensity FAD}}$

Definitions of fluorescence lifetime imaging parameters and the optical redox ratio, which are quantified for each cell.

#### 2.3.2 Manual cell segmentation

Manual immune and tumor cell segmentation was performed through a custom CellProfiler pipeline. Immune cells were segmented based on their mCherry intensity. Tumor cells were segmented based on their NAD(P)H intensity. For both cell types, single cells were circled and segmented as whole cells to include both nucleus and cytoplasm. The resulting segmented images were saved as masks. Using these masks and the raw imaging data from 2.3.1, fluorescence lifetime variables and redox ratio values were calculated for each individual cell. Calculations were performed using RStudio.

#### 2.3.3 Automated immune cell segmentation

Automated immune cell segmentation was performed through a custom Python based pipeline (**Figure S2**). Intensity and lifetime images of mCherry, thresholded to a predefined lifetime range of 800-1500 ps to reduce non-specific signal, were passed in as initial inputs to the pipeline. Initial global and local thresholding of intensity images through a series of standard thresholding methods – otsu, yen, min, triangle, local, and their combinations – was used to differentiate foreground vs. background (50, 51, 52). Next, a left-merge intersection was performed to combine the thresholded intensity image and the range-limited lifetime image. A canny edge detector was used to identify and label individual regions of interest (ROIs) in the resulting image. ROIs were expanded through a first round of binary dilation or closing to ensure proper coverage of cell bodies. Border clearing was performed to remove any partial cells followed by an edge detection step. A second round of binary dilation or closing was then performed. Following ROI generation, a small-item filter was used based on ROI area to limit noise and non-specific elements in the mask. Ensemble voting was performed *via* combinatorial analysis of intensity image thresholding and ROI region expansion methods. A maximum intensity projection was generated for each outcome, and a single ‘best’ binary mask was established by maximizing Dice coefficients compared against hand-segmented ground truth images for every image in the dataset. The segmentation and dilation procedures explained above resulted in whole cell immune cell masks that encompassed both nuclei and cytoplasm. These masks were qualitatively confirmed by visual inspection of whole cell segmentation. Whole cell masks were also quantitatively confirmed by performing mask dilations of 1, 2, and 3 pixels – which did not statistically differ (p>0.05) from the original masks for any fluorescence intensity or lifetime parameter. Final automated segmentation mask quality and accuracy was assessed through calculations of the Dice coefficient for each mask (**Table S1**). As needed, automated masks were also manually improved using Napari. Using these automated masks and the raw imaging data from 2.3.1, fluorescence lifetime variables and redox ratio values were calculated for each cell. Calculations of these single cell OMI parameters were performed using RStudio (Version 4.1.0).

### 2.4 Multiplex immunofluorescence

Excised tissues were formalin fixed and paraffin-embedded for antibody staining with a panel of fluorescent markers (CD4, mCherry, and CD8). Embedded sections were deparaffinized and hydrated prior to antigen retrieval and placement in blocking solution. Next, primary antibodies were sequentially applied upon removal of blocking solution at the following dilutions and incubation times: CD4 – 1:500 for 15 min, mCherry – 1:500 for 10 min, and CD8 –1:250 for 15 min. Secondary antibodies were then added following each primary antibody incubation using rat and rabbit secondary antibodies. The following staining dyes were added after secondary antibody washes at 1:100 dilution for 10 min: CD4 – Opal-dye 520, mCherry – Opal-dye 570, CD8 – Opal dye 620. Finally, stained sections were incubated in DAPI for 5 min at room temperature for nuclear labeling and mounted on coverslips for imaging. Imaging was performed at 40× using a Vectra multispectral imaging system (Akoya Biosciences) and a spectral library was generated to separate spectral curves for each of the fluorophores. Resulting images were analyzed using Nuance and inForm software (Akoya Biosciences).

### 2.5 Flow cytometry

Tumors and spleens were harvested from euthanized mice. Spleens were then dissociated into a single cell suspension and filtered with a 70 μm filter. Red blood cells were lysed using 1 mL ddH_2_O for 10 seconds, then spleen cells were stored in PBS on ice until aliquoted into flow cytometry tubes. Tumors were cut into ~1 mm chunks using surgical scissors. The tumor chunks were collected in gentleMACS C tubes (Miltenyi Biotec) containing 2.5 mL of RPMI 1640 + 10% fetal bovine serum, 100 U/mL penicillin, 100 μg/mL streptomycin, and 2 mM L-glutamine. 100μL of DNAse I solution in RPMI 1640 (2.5 mg/mL, Sigma-Aldrich) and 100 μL collagenase IV solution in RPMI 1640 (25 mg/mL, Gibco) were then added and the samples were run on a MacsQuant Octo Dissociator (Miltenyi Biotec) using the preset dissociation protocol for mouse tumor dissociations. After dissociation, the tumors were filtered through a 70 μm cell strainer, washed with 10 ml PBS, and stored on ice until being aliquoted in flow cytometry tubes.

Flow cytometry tubes containing 2-5x10^6^ tumor or spleen cells were labeled and stained with 0.5 μL GhostRed780 (Tonbo Biosciences) in 500μL of PBS per sample and light protected at 4°C for 20-30 minutes. Samples were then washed with flow buffer (PBS +2% FBS). F_c_ block was not used here. In the meantime, a master mix containing all antibody markers was prepared and aliquoted at 50 μL per flow tube (**Table S2**). Fluorescence minus one controls (FMOs) were also prepared for each marker minus live/dead. Cells were stained with surface markers for 30-45 min at 4°C in the dark. After staining, samples were washed in flow buffer and data acquired on an Attune™ NxT flow cytometer (Thermo Fisher) with manufacturer provided acquisition software. This instrument was maintained by the Flow Cytometry Core Lab through the University of Wisconsin Carbone Cancer Center, which performs daily quality control checks and instrument calibration using Attune™ Performance Tracking Beads (Thermo Fisher, cat 4449754). This cytometer was equipped with the following excitation lasers: 488nm (BL), 561nm (YL), 405m (VL), and 633nm (RL). The cytometer was equipped with the following channel/bandpass filter combinations: BL1 (530/30), BL2 (590/40), BL3 (695/40), YL1 (585/16), YL2 (620/15), YL3 (695/40), YL4 (780/60), VL1 (440/50), VL2 (512/25), VL3 (603/48), VL4 (710/50), RL1 (670/14), RL2 (720/30), and RL3 (780/60). Data were analyzed using FlowJo version 10.7.1 (FlowJo LLC, Becton Dickinson & Company (BD) 2006-2020). Gates were determined using FMOs. Gating strategies for each immune cell population are defined in **Table 2**.

**Table 2:** Flow cytometry gating strategies.

Population	Gating Definition (after Cells/Single/Live)
CD45 Lymphocytes	CD45+
CD8 T Cells	CD45+/CD3ϵ+/NK1.1-/CD8α+
CD4 T Cells	CD45+/CD3ϵ+/NK1.1-/CD4+
NKT Cells	CD45+/CD3ϵ+/NK1.1+
NK Cells	CD45+/CD3ϵ-/NK1.1+
B Cells	CD45+/CD3ϵ-/NK1.1-/CD19+
Macrophages	CD45+/CD3ϵ-/Ly6G-/F4/80+
Dendritic Cells	CD45+/CD3ϵ-/NK1.1-/CD11c+
Neutrophils	CD45+/CD3ϵ-/Ly6G+/CD11b+

Gating definition for flow cytometry analysis of each immune cell population.

### 2.6 Heatmaps

Z-score heatmaps were created using the Complex Heatmap package (RStudio) (53, 54). Hierarchical clustering of single cells was performed based on twelve OMI parameters (NAD(P)H τ_m_, τ_1,_ τ_2,_ α_1_, α_2_; FAD τ_m_, τ_1,_ τ_2,_ α_1_, α_2_; cell size; optical redox ratio) and calculated using Ward’s method. Labels for cell type, tissue type, and mouse were added afterwards and not included in cluster analysis.

### 2.7 Statistical analysis

Mann–Whitney statistical tests for non-parametric, unpaired comparisons were performed to assess differences in OMI parameters between cell types. This test was chosen because these data distributions were not assumed to be parametric. Results are represented as dot plots showing mean ± standard deviation where each dot represents a single cell (GraphPad Prism 9). Effect size was also calculated to assess differences in OMI parameters between cell types using Glass’s Δ because comparisons of very large sample sizes of individual cells almost always pass traditional significance tests unless the population effect size is truly zero (55). Glass’s Δ is defined as the difference between the mean of the experimental group (*M_1_*) and the mean of the control group (*M_2_*) divided by the standard deviation of the control group (*σ_control_*).


$$Glass^{\prime }s\;\Delta =\;\frac{M_{1\;}-M_{2}}{\sigma_{control}}$$

A Glass’s Δ > 0.8 was chosen to indicate significant effect size based on previous studies (22, 56, 57). When comparing immune cells from different tissues, the immune cells from the spleen were designated as the control. When comparing tumor cells and immune cells within the tumor, immune cells were designated as the control. Here, we report the absolute value of Glass’s Δ. The OMI heatmaps represent Z-score (4 standard deviations ± from the mean of all cells in the heatmap) for each OMI parameter per cell type (RStudio) (53). A Z-score of 0 indicates that data point score is identical to the mean score.

## 3 Results

### 3.1 CD4 mCherry reporter mouse demonstrates mCherry expression in all CD4 T cells as well as most CD8 T cells and NKT cells

**Figure 2:**
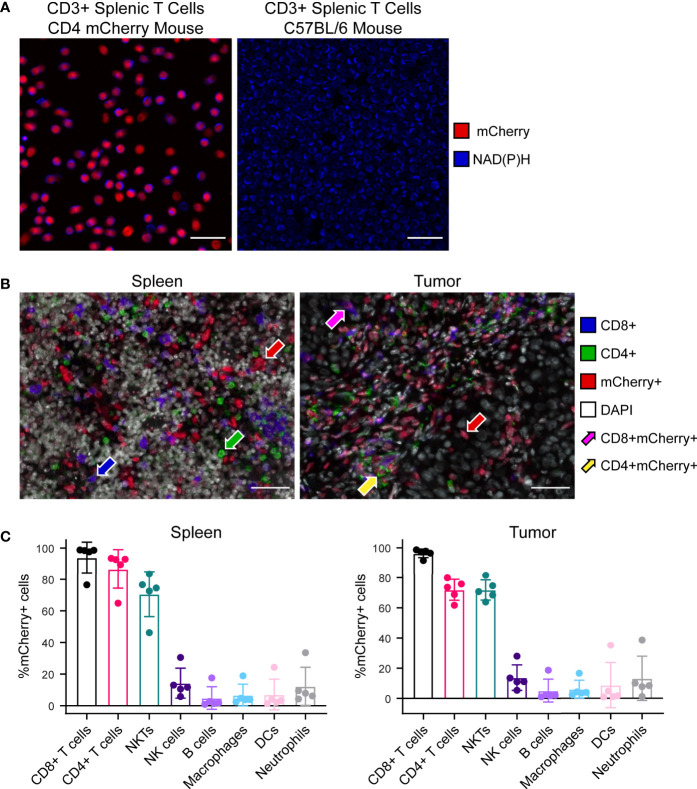
Characterization of CD4 mCherry reporter mouse tissues. **(A)** *Ex vivo* two-photon images of isolated CD3+ splenic T cells at 40× illustrate nuclear mCherry labeling of all T cell populations (red) within the reporter mouse (left) which colocalizes well with the metabolic coenzyme NAD(P)H also present in all T cells (blue), primarily in the cytoplasm. Within the unlabeled wild type C57BL/6 mouse (right), NAD(P)H but not mCherry is present in all T cells (blue). Cell density is higher for the wild type C57BL/6 image (right) compared to reporter mouse. Scale bar 25 μm. **(B)** Representative 40× multiplex immunofluorescence images from reporter mouse spleen (left) and B78 melanoma tumor (right) illustrates mCherry labeling of several subsets of immune cells. CD4+mCherry- cells with only green membrane (green arrow), CD8+mCherry- cells with only blue membrane (blue arrow), CD4-CD8-mCherry+ with only red nuclei (red arrow), CD4+mCherry+ with both red nuclei and green membrane (yellow arrow), CD8+mCherry+ with both red nuclei and blue membrane (magenta arrow). Scale bar 50 μm. **(C)** Quantitative cell population analysis by flow cytometry in both spleens (left) and B78 melanoma tumors (right) indicates efficient mCherry expression by the majority of CD4+ T cells (70-100%) with mCherry expression also occurring in other cells known to express CD4 during differentiation, including CD8 T cells and NKT cells (70-100%) as well as B cells, dendritic cells, and macrophages (<20%). The data also indicates very low mCherry expression by cells that do not typically express CD4 during differentiation, including NK cells and neutrophils (<20%) (n = 5 untreated mice, mean ± SD).

Multiphoton images of *ex vivo* isolated CD3+ splenic T cells illustrate successful nuclear mCherry-expression (red) by all T cell populations within the CD4 mCherry reporter mouse (**Figure 2A** left) with no mCherry present in the C57BL/6 wild type mouse (**Figure 2A** right). As expected, both reporter and wild type mice exhibit endogenous NAD(P)H (blue) in all T cells. Multiplex immunofluorescence images from reporter mouse spleen (**Figure 2B** left) and B78 tumor (**Figure 2B** right) illustrate mCherry-expression by several subsets of immune cells including CD4+mCherry+ (yellow arrow) and CD8+mCherry+ (magenta arrow) T cells as well as other immune cells. Quantitative cell population analysis *via* flow cytometry in reporter mouse spleens and B78 tumors (**Figure 2C**) indicates efficient mCherry-expression by all CD4+ T cells (70-100%), with mCherry labeling also occurring in other cells known to express CD4 during differentiation including CD8 T cells and NKT cells (70-100%) as well as B cells, dendritic cells, and macrophages (<20%). The data also indicates very low mCherry expression by cells that do not typically express CD4 during differentiation, including NK cells and neutrophils (<20%). During maturation, high CD4 expression is expected for CD4 and CD8 T cells as well as NKT cells while CD4 is expressed to a minor extent on macrophages, B cells, dendritic cells, and some monocytes (32–34). When comparing mCherry mean fluorescence intensity (MFI) of these more mature immune cell populations, CD8 T cells, CD4 T cells, and NKT cells exhibited the brightest mCherry signal with significantly lower MFI for all other populations (**Figure S3**). Additionally, the mCherry MFI of NK cells, B cells, macrophages, dendritic cells, and neutrophils overlapped with our negative control – a spleen from a wild type C57BL/6 mouse (**Figure S3**). Overall, the Cre-*lox* methods used to create this reporter mouse (**Figure S1**) were successful at labeling the majority of T cell and NKT populations known to express CD4 in either their mature state or during maturation. This reporter mouse model may be a useful tool to researchers performing tumor immunotherapy work where visualization of T cells *via* mCherry-expression is beneficial, especially for techniques where secondary methods can be used to further identify subsets of mCherry+ cells, if desired. If a highly specific CD4 T cell model is needed, this model may not be the optimal choice.

### 3.2 Intravital imaging enables concurrent visualization of tumor and immune cells

**Figure 3:**
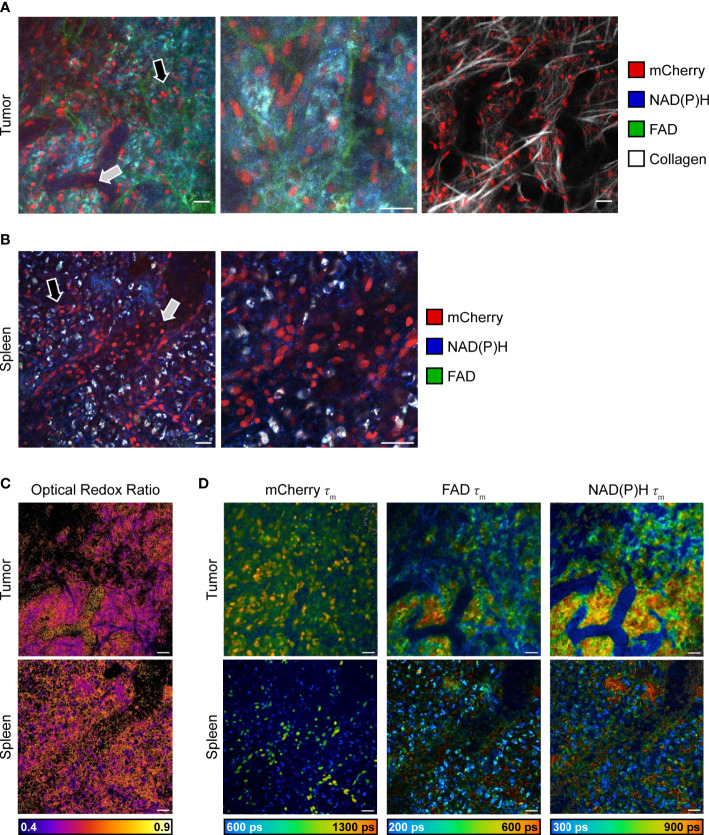
*In vivo* multiphoton images of immune and tumor cell populations. **(A)** Representative *in vivo* fluorescence intensity images of B78 melanoma growing in an untreated CD4 mCherry reporter mouse, live during skin flap surgery. Images (left and center) of all fluorophores show mCherry-expressing immune cells (red) infiltrating into tumor tissue (black arrow) and within vasculature (gray arrow) as well as autofluorescent NAD(P)H (blue) and FAD (green) expressed by all cells. Center image is a zoom of 2.0, inset from left image. Right image shows mCherry-expressing immune cells (red) interacting with collagen fibers (white) within the tumor microenvironment. Scale bar 25 μm. **(B)** Representative *ex vivo* fluorescence intensity images of spleen from untreated CD4 mCherry reporter mouse, spleen imaged immediately after euthanasia and excision. Images of all fluorophores show mCherry-labeled immune cells (red) within spleen tissue (black arrow) and vasculature (gray arrow) as well as autofluorescent NAD(P)H (blue) and FAD (green) expressed by all cells. Right image is a zoom of 2.0, inset from left image. Scale bar 25 μm. **(C)** Optical redox ratio images (the intensity of NAD(P)H divided by intensity of NAD(P)H plus FAD) show cellular redox balance within tumor (top) and spleen (bottom). Scale bar 25 μm. **(D)** Representative *in vivo* fluorescence lifetime images of B78 melanoma growing in an untreated CD4 mCherry reporter mouse. mCherry τ_m_ images show mCherry-expressing immune cells and their corresponding mean lifetime (τ_m_) values that are used to distinguish mCherry-expressing cells (typical τ_m_ = 1,300-1,500 ps) from nonspecific red autofluorescence *in vivo*. NAD(P)H and FAD τ_m_ images show NAD(P)H and FAD intensity and corresponding mean lifetime values that provide insight into tumor metabolism. ps, picoseconds. Scale bar 25 μm.

Intravital imaging of reporter mouse tumors was performed using a skin flap surgery method (**Figure 1**) to image B78 melanoma tumor and immune cell subsets live. Representative *in vivo* fluorescence intensity tumor images (**Figure 3A** left, middle) highlight mCherry-expressing immune cells (red) infiltrated into the tumor tissue and within the vasculature as well as autofluorescent NAD(P)H (blue) and FAD (green) expressed and detected in all cells. Collagen fibers (endogenous SHG signal, white) and their interaction with mCherry-expressing immune cells were also visualized within the TME, where some immune cells are co-localized with collagen fibers (**Figure 3A** right). *Ex vivo* imaging of freshly excised reporter mouse spleens was completed within one hour post mouse euthanasia, accurately capturing splenic metabolism based on our previous work that showed that *ex vivo* metabolism is statistically identical to *in vivo* metabolism for up to 12 hours post euthanasia (46). Representative *ex vivo* fluorescence intensity spleen images (**Figure 3B**) highlight mCherry-expressing immune cells (red) within the spleen tissue and lining the vasculature. Autofluorescent NAD(P)H (blue) and FAD (green) can be detected in all cells. Optical redox ratio images inform on tumor and immune cell redox balance in both tumor and spleen tissues (**Figure 3C**). Representative *in vivo* fluorescence lifetime images of the tumor and spleen show the mean lifetime of mCherry, NAD(P)H, and FAD mapped to their location with each cell (**Figure 3D**). The mCherry mean lifetime values (typical τ_m_ = 1,300-1,500 ps) help distinguish mCherry+ cells from nonspecific red autofluorescence *in vivo* (58–61). The NAD(P)H and FAD mean lifetime values inform on protein-binding and other environmental changes within the cell. Our imaging technology provides clear, concurrent images of both B78 and mCherry+ immune cells from both cancerous and healthy tissues. This imaging approach may be helpful to researchers performing immunotherapy, infectious disease, and autoimmune disorder research where the focus is on both the immune system and local tissues.

### 3.3 Automated immune cell segmentation algorithm provides fast, reproducible, single cell metabolic information

**Figure 4:**
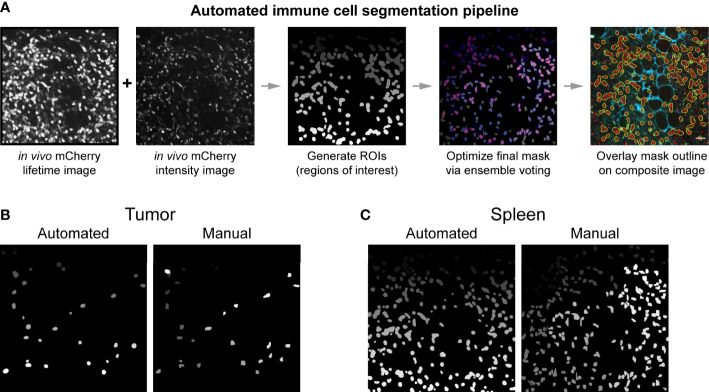
Automated immune cell segmentation pipeline to analyze *in vivo* multiphoton optical metabolic images. **(A)** Flowchart showing automated segmentation pipeline analysis on a single example image for mCherry-expressing immune cells. Pipeline developed in Python. This pipeline enables the extraction of metabolic data from each immune cell. **(B, C)** Representative examples of the comparison between an automated segmentation mask versus a manual segmentation mask of immune cells in a **(B)** B78 tumor (Mouse 2 FOV 7) and **(C)** spleen (Mouse 1 FOV6) field of view. Dice coefficient scores were also calculated to show quantitative accuracy between automated masks and manual masks, as shown in **Table S1**. All fields of view (FOV) 300 × 300 μm.

To obtain quantitative metabolic information from the images we acquired, single cell segmentation was performed. Traditionally, cell segmentation has been performed manually. However, *in vivo* and *ex vivo* images often have low signal to noise ratios, additional cell types that complicate analysis, and poorly distinguished cell boundaries – all of which makes the manual segmentation process difficult and slow. Manual segmentation also introduces variability between researchers. To promote faster and more reproducible segmentation, an automated single cell segmentation method was developed (**Figures 4A**, **S2**) to segment immune cells. This custom Python algorithm provided unbiased, accurate segmentation masks and validated results against hand segmented ground truth masks (**Figures 4B, C**). The automated segmentation algorithm performed the segmentation in a fraction of the time required to segment the images manually – on the order of seconds compared to hours. Additionally, this automated method combined both fluorescence intensity and fluorescence lifetime images to create the most accurate immune cell mask – a step that helps differentiate true mCherry signal from nonspecific red autofluorescence in the tissue. The algorithm also allowed rapid iteration of cell segmentation to provide the most optimized mask. The final immune cell masks were high quality and closely matched the manually segmented masks (**Table S1**). Dice coefficient accuracy metrics were calculated to compare images, where Dice scores range from 0 (no overlap) to 1 (perfect match). The image segmentation field typically considers 0.5 the minimum acceptable Dice coefficient, with higher regard for segmentation techniques that produce Dice coefficients in the 0.6-0.7 range (62–64). Our automated segmentation algorithm performed better on images from the spleen (Dice coefficients average 0.663) compared to images from the tumors (Dice coefficients average 0.590). Immune cell size was more heterogenous and signal to noise was worse within the tumor images, likely contributing to this difference in performance.

### 3.4 Optical metabolic imaging distinguishes immune cells from healthy and cancerous tissue origin

**Figure 5:**
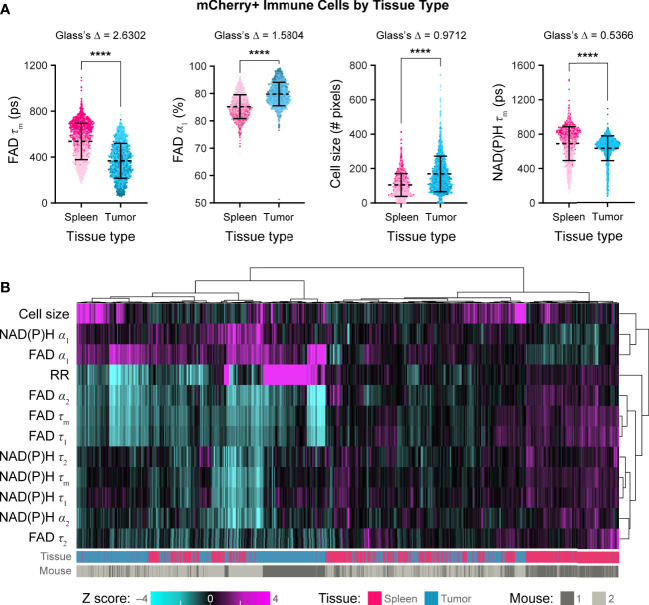
*In vivo* optical metabolic imaging of mCherry+ immune cells within tumor and spleen discriminates metabolic changes based on tissue type. **(A)** Single cell mCherry+ immune cell OMI parameters from spleen (n = 1672 cells) and B78 tumor (n =1688 cells) across n = 2 mice. Immune cells from B78 tumors exhibit significantly decreased FAD τ_m_ (361 ps vs. 554 ps) and significantly increased FAD α_1_ (90% vs. 85%) compared to immune cells from spleens (Glass’s Δ >0.8). Immune cells from B78 tumors are significantly larger in size (149 pixels vs. 95 pixels) compared to immune cells from spleens (Glass’s Δ >0.8). Immune cells from B78 tumors exhibit decreased NAD(P)H τ_m_ (667 ps vs. 734 ps) compared to immune cells from spleens; though the p value indicates substantial significance (p<0.0001), only a trend is observed using the more conservative Glass’s Δ test (Glass’s Δ = 0.5366). Data points colored to reflect individual replicates acquired on different days. Bars indicate mean ± SD, Mann-Whitney U Test, **** p<0.0001 shown for reference; all Mann-Whitney U Test comparisons are significant due to the large sample size so Glass’s Δ was also included as a more conservative significance test, see Methods. **(B)** Heatmap of z-scores of 12 OMI parameters from immune cells within both spleens and tumors. Hierarchical clustering of single cells indicates data clusters best by tissue type. Each column is a single cell (n = 3360 cells).

Using these techniques, we then quantified single cell mCherry+ immune cell OMI parameters from spleens and B78 tumors. Immune cells from B78 tumors exhibited significantly decreased FAD τ_m_ compared to immune cells from spleens (**Figure 5A**, Glass’s Δ > 0.8). Immune cells from B78 tumors also exhibited significantly increased FAD α_1_, the proportion of protein-bound FAD, compared to immune cells from spleens (**Figure 5A**, Glass’s Δ > 0.8). Additionally, immune cells from B78 tumors are significantly larger in size compared to immune cells from spleens (**Figure 5A**, Glass’s Δ > 0.8). Finally, immune cells from B78 tumors exhibited decreased NAD(P)H τ_m_ compared to immune cells from spleens; though the p value indicates substantial significance (p<0.0001), only a trend is observed using the more conservative Glass’s Δ test (**Figure 5A**, Glass’s Δ = 0.5366). We also visualized single cell heterogeneity based on OMI parameters from immune cells within spleens and tumors using a heatmap of z-scores where each column is a single immune cell (**Figure 5B**). The OMI parameters cluster cells best by tissue type (spleen vs. tumor) with some clustering by mouse. When the OMI parameters are plotted without the optical redox ratio, we continue to see consistent clustering by tissue type (**Figure S4**). Taken together, these data show that *in vivo* OMI can distinguish immune cells from different tissues of origin and visualize single cell heterogeneity in metabolism.

### 3.5 Optical metabolic imaging distinguishes tumor and infiltrating immune cells within the same tumor

**Figure 6:**
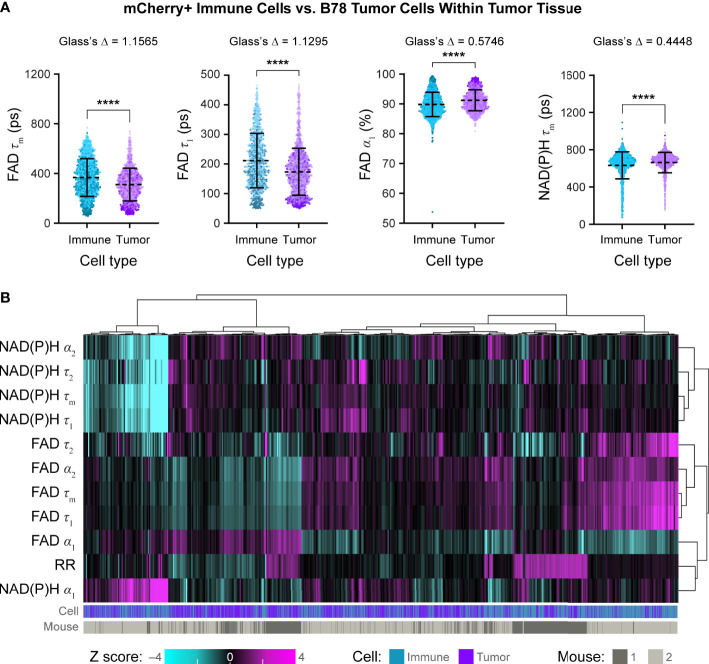
*In vivo* optical metabolic imaging within tumors discriminates infiltrating immune and tumor cells. **(A)** Single cell OMI parameters from infiltrating mCherry+ immune cells (n = 1688 cells) and B78 tumor cells (n =1455 cells) within the tumors from n = 2 mice. Immune cells from the B78 tumors exhibit significantly increased FAD τ_m_ (361 ps vs. 306 ps) and FAD τ_1_ (213 ps vs. 174 ps) compared to B78 tumors cells (Glass’s Δ > 0.8). Immune cells from B78 tumors exhibit decreased FAD α_1_ (89% vs. 91%) compared to B78 tumor cells; though the p value indicates substantial significance (p<0.0001), only a trend is observed using the more conservative Glass’s Δ test (Glass’s Δ = 0.5746). Immune cells from B78 tumors exhibit decreased NAD(P)H τ_m_ (667 ps vs. 686 ps) compared to B78 tumor cells; though the p value indicates substantial significance (p<0.0001), only a trend is observed using the more conservative Glass’s Δ test (Glass’s Δ = 0.4448). Data points colored to reflect individual replicates acquired on different days. Bars indicate mean ± SD, Mann-Whitney U Test, ****p<0.0001 shown for reference; all Mann-Whitney U Test comparisons are significant due to the large sample size so Glass’s Δ was also used as a more conservative significance test, see Methods. **(B)** Heatmap of z-scores of 12 OMI parameters from immune cells and tumor cells within B78 tumors. Hierarchical clustering of single cells indicates data clusters best by mouse with some clustering by cell type. Each column is a single cell (n = 3143 cells).

We also quantified single cell OMI parameters from B78 tumor cells versus tumor infiltrating mCherry+ immune cells within the same tumor. Tumor infiltrating immune cells exhibited significantly increased FAD τ_m_ compared to B78 tumor cells within the same tumor (**Figure 6A**, Glass’s Δ > 0.8). Tumor infiltrating immune cells also exhibited significantly increased FAD τ_1_, the lifetime of protein-bound FAD, compared to B78 tumor cells within the same tumor (**Figure 6A**, Glass’s Δ > 0.8). Additionally, tumor infiltrating immune cells exhibited a decreased FAD α_1_ and NAD(P)H τ_m_ compared to B78 tumor cells; though the p values both indicate substantial significance (p<0.0001), only a trend is observed using the more conservative Glass’s Δ test (**Figure 6A**, FAD α_1_ Glass’s Δ = 0.5746, NAD(P)H τ_m_ Glass’s Δ = 0.4448). We visualized single cell heterogeneity based on OMI parameters from tumor infiltrating immune cells and B78 tumor cells within the same tumors using a heatmap of z-scores where each column is a single cell (**Figure 6B**). The OMI parameters cluster cells best by mouse (mouse 1 vs. mouse 2) indicating variability in the different tumor microenvironments in the two mice. We also observed heterogeneity in OMI between the two tumors that were imaged on different days, which was especially prominent within the optical redox ratio parameter. Interestingly, when the OMI parameters are plotted without the optical redox ratio, cells cluster based on both the mouse and cell type (tumor infiltrating immune cell vs. tumor cell) (**Figure S5**). Taken together, these data show that *in vivo* OMI can distinguish tumor infiltrating immune cells from B78 tumor cells, all within the same tumor environment, and visualize single cell heterogeneity in metabolism.

## 4 Discussion

Metabolic reprogramming plays an active role in cancer progression (1, 2). Complex metabolic changes occur in parallel for both tumor and infiltrating immune cell populations in the TME. To improve clinical cancer outcomes, an improved understanding of tumor and immune cell metabolism and their interplay *in vivo* is greatly needed. Here, we demonstrated the potential power of intravital OMI to help fulfill this need by imaging single cell metabolism in tumor and immune cells *in vivo*.

Our mCherry reporter mouse model enabled *in vivo* tracking and segmentation of single immune cells. We showed that this immunocompetent mouse model had efficient mCherry-expression in nearly all T cells (CD4 and CD8) and NKT cells as well as a few other immune cell subsets *via* multiphoton imaging, multiplex immunofluorescence, and flow cytometry assays. Immune cell mCherry-expression was also shown to be consistent between spleen and B78 melanoma tumor tissues. The low mCherry expression observed on NK cells and neutrophils, which do not typically express CD4 during differentiation, is likely due to low levels of baseline, non-targeted mCherry expression in the floxed H2B-mCherry strain (Jax 023139) prior to introduction of Cre, which has been reported by Jackson Labs. Additionally, the mCherry reporter allowed simultaneous detection of NAD(P)H and FAD autofluorescence *in vivo* without fluorescence interference from the reporter. This reporter mouse model may be a useful tool to researchers performing tumor immunotherapy studies where visualization of CD3+ immune cells (such as CD4 T, CD8 T, or NKT cells) *via* mCherry-expression is beneficial.

*In vivo* OMI provided clear visualization of tumor and immune cells within both spleen and tumor tissues. We observed tumor infiltrating immune cells, immune cells within and lining blood vessels, and immune cells co-localized with collagen fibers. We captured autofluorescence changes within the healthy and diseased tissue context and visualized single cell heterogeneity in metabolism within immune cells and tumor cells. We note that the FAD channel includes contributions from other endogenous fluorophores in cells [*e.g.*, lipofuscin, flavin mononucleotide (FMN)] and the extracellular matrix (*e.g.*, collagen, elastin). The extracellular matrix signals from fibers are easily segmented from cells based on location and morphology. We do not anticipate that lipofuscin and FMN strongly affect our FAD signal changes because metabolic perturbations (cyanide) confirm expected changes in FAD intensity and lifetime. Additionally, the lifetimes of FMN and lipofuscin are longer and shorter, respectively, than the FAD lifetime observed in this study and in prior works (15, 65–68). Therefore, we label this the FAD channel to reflect its presence while acknowledging the caveat of other sources of contrast. Taken together, this *in vivo* imaging platform is a valuable technique to probe metabolic changes within intact, unlabeled tissues that better preserves the TME.

We also reported an automated segmentation algorithm that provided robust segmentation of single immune cells from our *in vivo* images. The algorithm incorporated both fluorescence intensity and lifetime information for more accurate cell segmentation while dramatically reducing segmentation time (seconds vs. hours for automated vs. manual segmentation). Dice coefficients to compare the accuracy of the automated segmentation algorithm to manual segmentation showed robust results, with the spleen images providing the highest agreement. Overall, this automated segmentation algorithm enabled single cell segmentation and quantification of metabolism within mCherry-expressing immune cells *in vivo* and *ex vivo*.

Optical metabolic imaging parameters discriminated immune cells from spleen and cancerous tissues of origin. Immune cells from B78 tumors are larger with an increase in protein-binding of FAD and decreased FAD τ_m_ and NAD(P)H τ_m_ compared to immune cells in spleens. We have previously shown that T cell activation correlates with a decrease in FAD τ_m_ and NAD(P)H τ_m_ along with an increase in FAD α_1_ (27), as we also showed here, providing confidence in our findings for immune cells from B78 tumors *in vivo*. Further, our observed decrease in FAD τ_m_ in immune cells within the tumor compared to spleen trends the same as our previously reported work with macrophages in tumors vs. skin (26). Additionally, the observed decrease in FAD τ_m_ found in immune cells within B78 tumors versus spleens may be partly due to decreased pH within the TME which has been shown to influence autofluorescence lifetime (69, 70). We also observed a significant increase in immune cell size from B78 tumors compared to spleens. This size difference is expected as immune cell size typically increases during stimulation, activation, and proliferation, which can occur within the tumor microenvironment (27, 71–74). Our analysis of single cell heterogeneity based on all OMI parameters showed clustering of immune cells based on tissue of origin – spleen versus tumor. Therefore, our metabolic imaging is sufficient to distinguish immune cell population features based on size and metabolic differences within the TME compared to spleen.

Finally, metabolic imaging parameters also showed differences between immune cells and tumor cells within the same tumor environment. We found that tumor infiltrating immune cells exhibited an increase in FAD τ_m_ and lifetime of protein-bound FAD (FAD τ_1_) with a decrease in amounts of protein-bound FAD (FAD α_1_) and NAD(P)H τ_m_ compared to the B78 tumors cells within the same tumor. This increase in FAD τ_m_ in non-malignant, infiltrating immune cells compared to B78 tumor cells trends the same as other reported work comparing FAD lifetime of non-malignant breast cells vs. breast cancer cells (17). We also observed a significant increase in the lifetime of protein-bound FAD (FAD τ_1_) for tumor infiltrating immune cells compared to B78 tumor cells. The fluorescence lifetime of protein-bound FAD changes with varying NAD+ concentrations due to increased FAD quenching in the presence of NAD+. So, this increase in protein-bound FAD may be indicative of a decrease in NAD+ concentration in infiltrating immune cells versus tumor cells (13, 75). Similarly, prior work has shown a comparable decrease in the NAD(P)H τ_m_ of macrophages compared to breast cancer cells (24) and non-malignant brain cells compared to glioblastoma (76). Taken together, tumor infiltrating immune cells exhibited an increase in FAD τ_m_ and lifetime of protein-bound FAD with a decrease in amounts of protein-bound FAD and NAD(P)H τ_m_ – which may be indicative of a shift towards a more glycolytic metabolism. These findings are in trend with prior work where it has been widely reported that many cancer cells primarily metabolize glucose using glycolysis (3–7). Heterogeneity analysis of all OMI parameters in immune and tumor cells within the same melanoma tumors showed clustering by mouse – highlighting the heterogeneity that occurs within each unique TME, even though these were mice from the same strain (nearly genetically identical), and each was implanted with the exact same B78 melanoma cells. However, when the OMI parameters are plotted without the optical redox ratio, cells cluster based on mouse and cell type (tumor infiltrating immune cell vs. tumor cell), indicating that the fluorescence lifetime parameters can capture differences in the metabolism of the immune vs. tumor cells. The optical redox ratio is an intensity-based measurement with increased sensitivity to day-to-day changes in laser power, laser alignment, tissue autofluorescence differences, and tissue composition differences such as increased presence of red blood cells. These factors make the redox ratio more sensitive to artifacts compared to fluorescence lifetime measurements that are self-referenced. Some of the observed immune cell metabolic heterogeneity may be partly attributed to the presence of three main mCherry+ immune cell types composing this group: CD4 T cells, CD8 T cells, and NKT cells. As these cells play different roles, their metabolic phenotypes also differ especially in the context of the TME. For example, activated CD4 T cells have been shown to rely more heavily on oxidative phosphorylation compared to their CD8 T cell counterparts in mice (77, 78). Similarly, CD8 T cells have been shown to be less sensitive to oxygen changes, which may result in differences compared to CD4 T cells within the hypoxic TME (79, 80). In contrast, NKT cells suppress glycolysis in the TME and rely on enhanced oxidative phosphorylation for function (81, 82). Heterogeneity within the TME itself may also contribute to the observed immune and tumor cell metabolic heterogeneity, influenced by factors such as pH as well as nutrient and oxygen availability. For example, tumor cells that are closer in proximity to blood vessels – resulting in higher oxygen concentrations and more nutrients – contain higher mitochondrial content, exhibit increased mitochondrial respiration, have a higher optical redox ratio, and exhibit a more aggressive phenotype compared to tumor cells far away from blood vessels (83–85). Immune cells are also affected by environmental factors, for example, more acidic pH (often due to an increase in lactate) in the TME inhibits mobility and tumor infiltration of both CD4 and CD8 T cells (86, 87). These factors, and many more, contribute to the metabolic heterogeneity we observed within our tumor and immune cell populations. Overall, we showed the versatility of *in vivo* OMI, our mCherry reporter mouse, and single cell segmentation to study single cell metabolic heterogeneity within different tissues and cell types of interest in the context of melanoma.

Although *in vivo* OMI provides information on metabolic changes across cells and tissues *in vivo*, alone it is not sufficient to specify a biological mechanism. Combining our live, spatial imaging with additional assays such as metabolite measurements *via* mass spectrometry or radiolabel tracing of glucose should identify specific metabolic pathways and possible drug targets (88–91). There is also great potential to use this *in vivo* imaging platform to assess drug, and especially immunotherapy, treatments *in vivo*, work that we are currently pursuing (40–42). Future studies may include the analyses done here – in mice receiving immunotherapy regimens known to be effective compared to those from which tumors are resistant or escape – in combination with other analyses of immune cell function, phenotype, and gene expression to identify metabolic patterns characteristic of effective anti-tumor immunity. We note that though only two animals were imaged in this study, single cell statistical power was high (3360 immune cells, 1455 tumor cells). In future studies focused on biological endpoints such as immunotherapy regimens, we will include additional animals. Ultimately, this imaging approach could provide insight into tumor and immune cell metabolism in a label free, *in vivo* manner to develop more effective cancer therapies.

## Data availability statement

The original contributions presented in the study are included in the article/**Supplementary Material**, further inquiries can be directed to the corresponding author/s. The code presented in this study is available and deposited on GitHub at the following link: https://github.com/skalalab/heaton_a-automated_immune_cell_segmentation.

## Ethics statement

The animal study was reviewed and approved by Institutional Animal Care and Use Committee at University of Wisconsin-Madison.

## Author contributions

AHe designed the experiments, collected the data, designed and performed the analysis, interpreted the data, generated the figures, and drafted the manuscript for this study. PR designed and performed the analysis and assisted in editing the manuscript. AHo assisted in collecting the data, designing and performing the analysis, and editing the manuscript. AL assisted in performing the analysis. AE assisted in designing the experiments, interpreting the data, and editing the manuscript. PS and MS assisted in designing the experiments and analysis, interpreting the data, and editing the manuscript. All authors contributed to the article and approved the submitted version.
