# Supplementary material for: Single cell metabolic imaging of tumor and immune cells in vivo in melanoma bearing mice
Source: Front Oncol. 2023 Mar 20;13:1110503. doi: 10.3389/fonc.2023.1110503 (PMC10067577; doi:10.3389/fonc.2023.1110503)
Supplement: Supplementary file 1 [file DataSheet_1.pdf]

Supplementary Material

1 Supplementary Figures

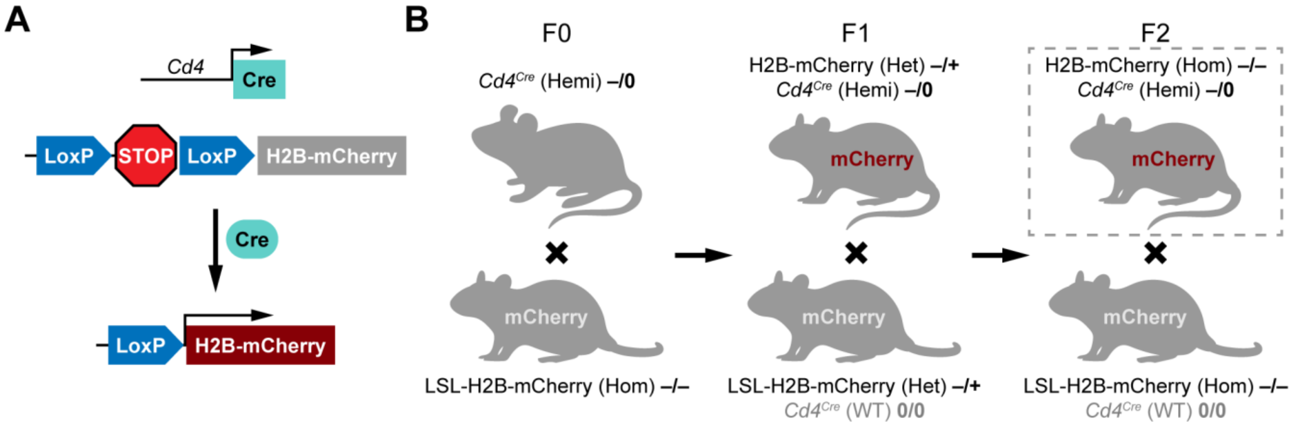

Supplementary Figure 1. CD4 mCherry reporter mouse model generation. (A) Genetic construct for CD4 mCherry reporter mice by Cre-*lox* recombinase crossing. A floxed H2B-mCherry mouse was crossed with a CD4-Cre mouse leading to Cre recombinase, removal of the stop codons, and CD4 targeted expression of mCherry. **(B)** Breeding scheme to produce experimental mice. First, a CD4-Cre hemizygous mouse was crossed with a Lox-Stop-Lox (LSL)-H2B-mCherry homozygous mouse to produce F1 offspring. Second, an H2B-mCherry heterozygous/CD4Cre hemizygous mouse was crossed with a LSL-H2B-mCherry heterozygous/CD4Cre wild type mouse to produce F2 offspring. H2B-mCherry homozygous/CD4Cre hemizygous mice were used for all experiments here (F2, gray dashed box). Additional offspring produced that are not shown in this scheme. Homozygous gene expression indicated by -/- symbols. Heterozygous gene expression indicated by -/+ symbols. Hemizygous gene expression indicated by -/0 symbols. No gene expression or wild type indicated by 0/0 symbols.

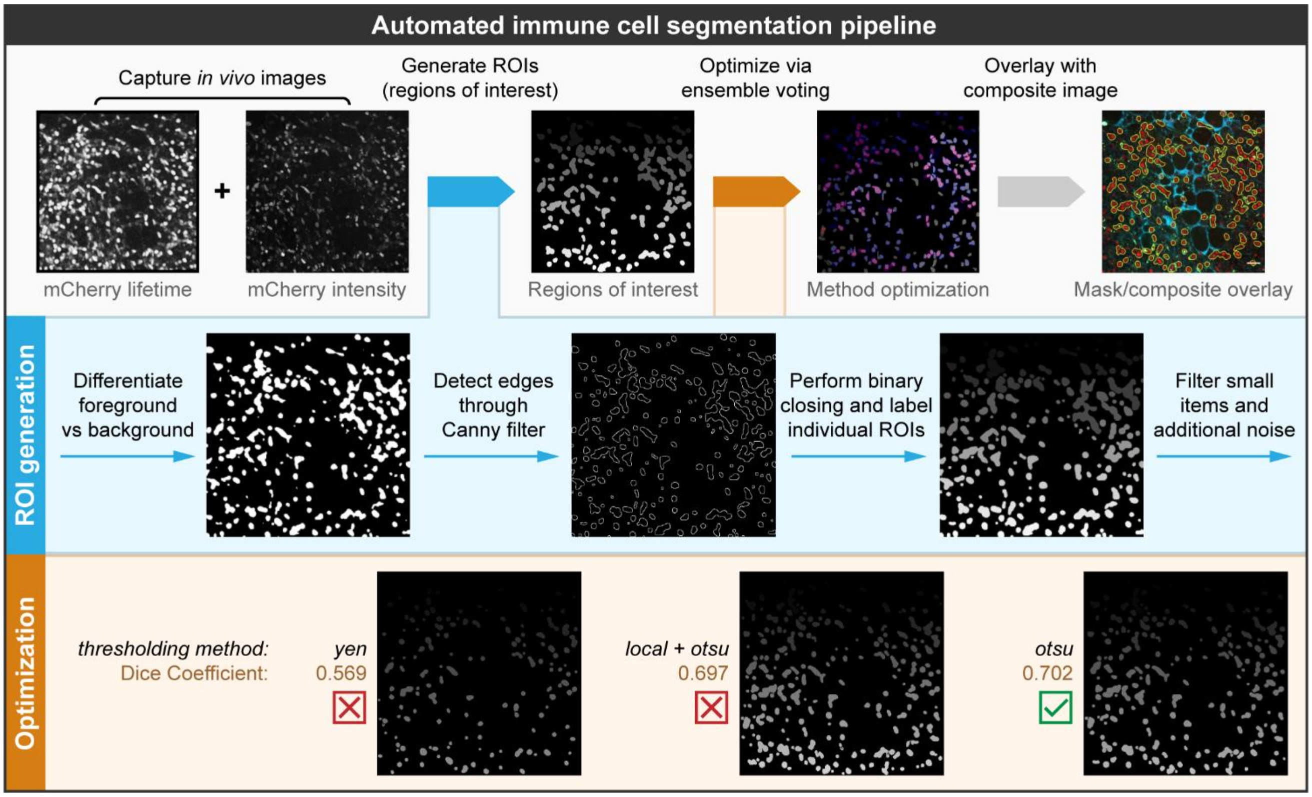

Supplementary Figure 2. Detailed flowchart of automated immune cell segmentation pipeline. Flowchart showing additional details of the automated segmentation pipeline for mCherry-expressing immune cells, developed in Python. This pipeline enables the extraction of autofluorescence features from each immune cell. Details on the generation of regions of interest (ROIs) are provided in the blue row, and details on the ensemble voting procedure to select the optimal thresholding method are provided in the orange row.

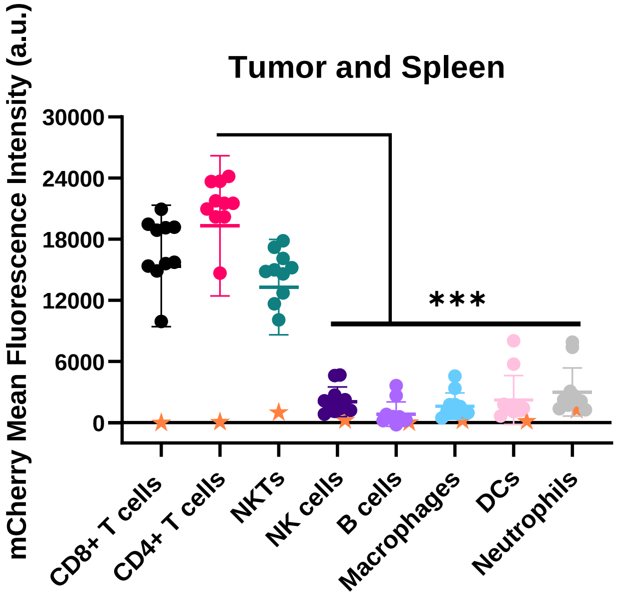

Supplementary Figure 3. Flow cytometry mCherry mean fluorescence intensity values for spleen and tumor immune cell populations. Cell population analysis by flow cytometry for combined spleen and B78 melanoma tumor samples. mCherry mean fluorescence intensity (MFI) across all immune cell types indicates CD8 T cells, CD4 T cells and NKT cells exhibit the highest mCherry MFI. In contrast, mCherry MFI for NK cells, B cells, macrophages, dendritic cells, and neutrophils are significantly lower than the CD4 T cell MFI (***p<0.001) and very comparable to the MFI from the negative control (orange star), a spleen from a C57BL/6 wild type mouse with no mCherry (n = 6 untreated mice total: n = 5 mCherry reporter mice, n = 1 C57BL/6 Jax wild type mouse, mean ± SD, Mann-Whitney U Test).

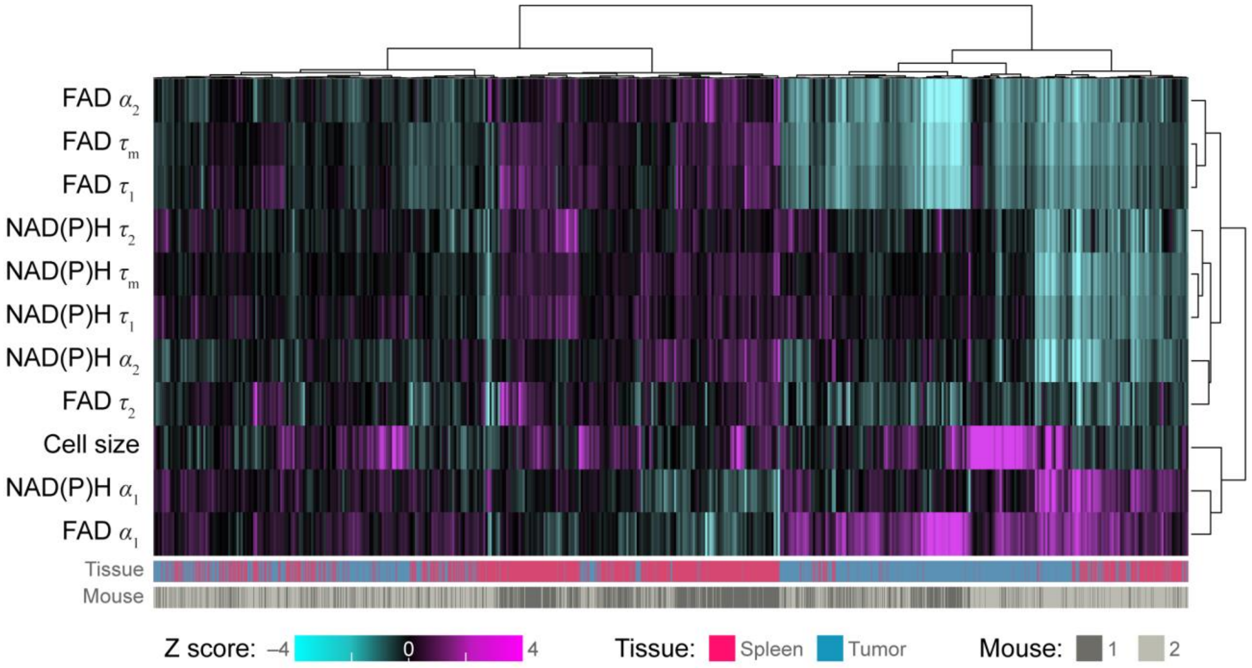

Supplementary Figure 4. Heatmap of *in vivo* optical metabolic imaging parameters of mCherry+ immune cells within tumor and healthy spleen discriminates metabolic changes based on tissue type. Heatmap of z-scores of 11 OMI parameters from immune cells within both spleens and tumors, with optical redox ratio removed. Hierarchical clustering of single cells indicates data clusters best by tissue type. Each column is a single cell (n = 3360 cells).

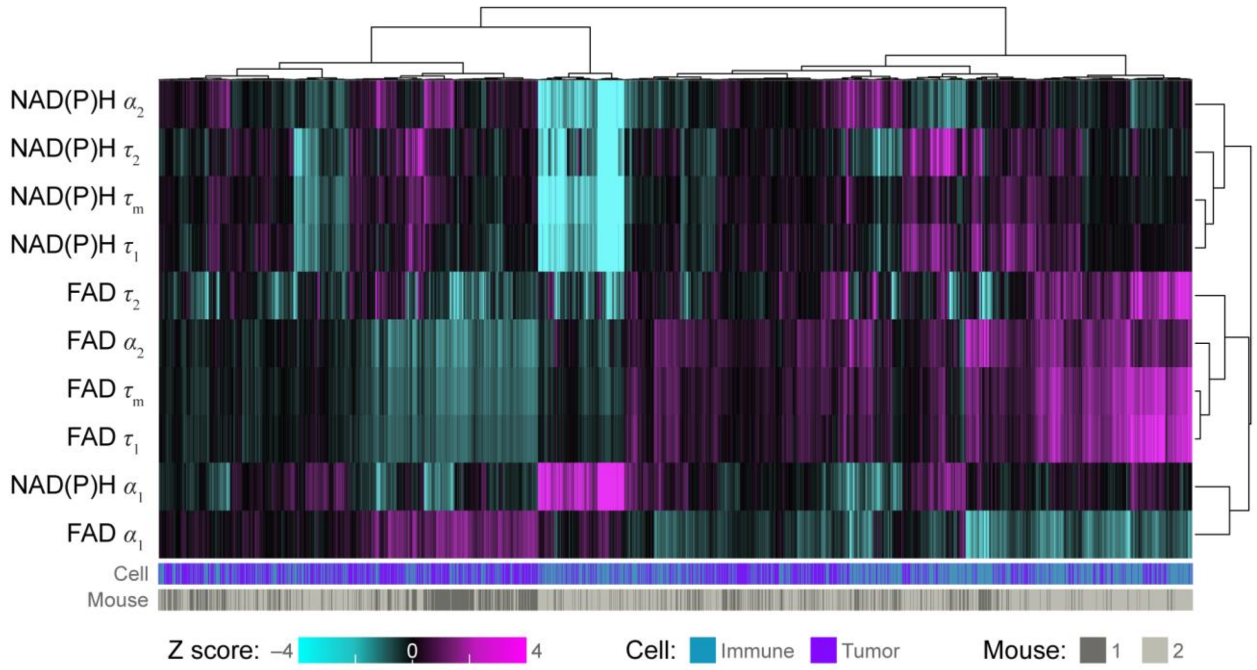

Supplementary Figure 5. Heatmap of *in vivo* optical metabolic imaging parameters of tumors discriminates infiltrating immune and tumor cells. Heatmap of z-scores of 11 OMI parameters from immune cells and tumor cells within B78 tumors, with optical redox ratio removed. Hierarchical clustering of single cells indicates data clusters by both mouse and cell type. Each column is a single cell (n = 3143 cells).

2 Supplementary Tables

$$\text{Dice } Coefficient = \frac{2 \times area\ of\ overlap\ between\ masks}{total\ number\ of\ pixels\ from\ both\ masks}$$

Mouse 1				Mouse 2			
B78 Tumor		Spleen		B78 Tumor		Spleen	
FOV	Dice Coefficient	FOV	Dice Coefficient	FOV	Dice Coefficient	FOV	Dice Coefficient
1	0.444	1	0.658	1	0.696	1	0.605
2	0.599	2	0.620	2	0.605	2	0.646
3	0.544	3	0.710	3	0.374	3	0.667
4	0.617			4	0.729	4	0.626
5	0.632			5	0.340		
6	0.643			6	0.723		
7	0.643			7	0.630		
8	0.678			8	0.512		
Average across all FOV	0.590	Average across all FOV	0.663	Average across all FOV	0.580	Average across all FOV	0.636

Supplementary Table 1. Accuracy of automated immune cell segmentation versus manual segmentation. The Dice coefficient compares the accuracy of our automated segmentation approach versus manual immune cell segmentation using CellProfiler pipelines. Dice coefficient scores range from 0 indicating no overlap in the masks, to 1 indicating identical masks. FOV, field of view.

Antibody	Fluorophore	Clone	Company	Catalog #	μL / test
CD45	BV510	30-F11	BioLegend	103138	1
CD4	FITC	GK1.5	Tonbo Biosciences	35-0041-U500	1.5
CD8α	BV711	53-6.7	Biolegend	100747	1
CD3ε	PE-Cy5	145-2C11	BioLegend	100310	1
CD11b	BB700	M1/70	BD Biosciences	566416	1
CD11c	AF700	N418	BioLegend	117319	2
CD19	APC	6D5	BioLegend	115512	1
Ly6G	PE	1A8	BioLegend	127608	1
NK1.1	BV605	PK136	BioLegend	108739	1.3
F4/80	PE-Cy7	BM8	BioLegend	123114	1
Live/Dead	GhostRed780		Tonbo biosciences	13-0865-T500	0.5
	mCherry	Transgenic CD4 mCherry reporter mouse			

Supplementary Table 2. Flow cytometry antibody information. Antibody specifications and quantities for flow cytometry.
